# Extracellular Matrix: Emerging Roles and Potential Therapeutic Targets for Breast Cancer

**DOI:** 10.3389/fonc.2021.650453

**Published:** 2021-04-22

**Authors:** Yunchun Zhao, Xiaoling Zheng, Yongquan Zheng, Yue Chen, Weidong Fei, Fengmei Wang, Caihong Zheng

**Affiliations:** ^1^ Department of Pharmacy, Women’s Hospital, School of Medicine, Zhejiang University, Hangzhou, China; ^2^ Key Lab Women’s Reproductive Health, Women’s Hospital, School of Medicine, Zhejiang University, Hangzhou, China

**Keywords:** extracellular matrix, breast cancer, therapeutic targets, nanoparticles, remodeling enzymes

## Abstract

Increasing evidence shows that the extracellular matrix (ECM) is an important regulator of breast cancer (BC). The ECM comprises of highly variable and dynamic components. Compared with normal breast tissue under homeostasis, the ECM undergoes many changes in composition and organization during BC progression. Induced ECM proteins, including fibrinogen, fibronectin, hyaluronic acid, and matricellular proteins, have been identified as important components of BC metastatic cells in recent years. These proteins play major roles in BC progression, invasion, and metastasis. Importantly, several specific ECM molecules, receptors, and remodeling enzymes are involved in promoting resistance to therapeutic intervention. Additional analysis of these ECM proteins and their downstream signaling pathways may reveal promising therapeutic targets against BC. These potential drug targets may be combined with new nanoparticle technologies. This review summarizes recent advances in functional nanoparticles that target the ECM to treat BC. Accurate nanomaterials may offer a new approach to BC treatment.

## Introduction

According to global cancer data, the incidence and mortality rates of breast cancer (BC) in women in 2019 were 24.20% and 15.00%, respectively, and both ranked first among female malignant tumors. Despite significant advances in the diagnosis and early treatment of BC, this malignant tumor still accounts for more than 600,000 deaths annually. Most cancer-related deaths are associated with cancer cells metastasizing to other sites of the body and causing new tumors in secondary organs (1). Although early BC has good prognosis after treatment, controlling cancer metastasis in clinical settings remains challenging (2, 3). There is an urgent need to better understand the mechanisms of metastasis and exploit novel strategies to prevent and treat advanced BC.

The extracellular matrix (ECM) is an important component of cellular biology (4). Proteins in the ECM include collagen, laminin (LN), fibronectin (FN), hyaluronan, and matricellular proteins that serve as structural scaffolds to maintain tissue integrity and sustainability (5). The ECM is crucial for embryonic development, production of new tissue structures, and maintenance of human tissues and homeostasis (6, 7). However, the ECM is not just a simple framework; it is a highly dynamic and complex network of molecules surrounding cells within the tissue. ECM components provide biochemical and biomechanical contexts for cells and have received increasing attention for their important biological roles in BC progression and metastasis (8). Evidence suggests that ECM components are dynamic during cancer progression and may promote metastatic spread (9). Regulation of key ECM components *via* targeting of matrix-mediated pre-tumorigenic signals or *via* promotion of tumor suppressive signals may be a promising strategy to address BC invasion and metastasis (10).

Nanoparticles (NPs) are widely used in biomedical research and are increasingly applied in oncologic research because of their excellent physicochemical properties (11). They generally consist of an outer shell and an inner core of buried drugs or proteins (12). Myriad NPs are used in antitumor therapy; liposomes, micelles, polymers, metal NPs, viral NPs, antibodies, and dendrimers have been widely applied in biological applications, including as drug carriers, tumor monitors, and cell markers (11, 12). Abnormalities of some receptors, enzymes and other components in the ECM allow breast tumors to be distinguished from the normal mammary gland. Specific metabolic pathways and rapid tumor proliferation lead to pathological blood vessel formation, low pH, low oxygen tension, and high interstitial pressure—aspects that are fundamental to the design of NP drug delivery systems (13). ECM serves as the soil for tumor cells to grow in, but it also contains complex factors to interfere with tumor progression and prognosis. By re-educating the ECM, the tumor constructs a micro-ecosystem to develop itself, escape immune attack, and even resist exogenous injury. NPs based on ECM regulation have been extensively studied because of their ability to accurately target tumor ECM components and reverse tumor progression (14–17).

In this review, we discuss the roles for ECM in the development and metastasis of BC, including ECM composition, molecular mechanisms related to ECM dynamics and remodeling in cancer, therapy resistance, and potential therapeutic targets. We also highlight ECM-regulated nanotherapeutic strategies, including degradation of the tumor ECM, mimic of the ECM to inhibit tumor progression, and alteration of the ECM fabrication as approaches to efficient BC suppression. Finally, we consider the future expectations for and challenges of ECM-targeted nanotherapeutics for clinical cancer therapy.

## ECM Composition in BC Progression

Increasing evidence indicates that the ECM composition continues to change during BC progression and may promote metastatic spread. The morphology, physical strength, biochemical characteristics, and other parameters of the ECM in BC differ from the ECM of normal breast tissue (18–20). ECM components can be divided into three groups: (1) structural proteins, such as collagen and elastin, that give the ECM strength and toughness; (2) glycosaminoglycans (GAGs) and proteoglycans that can form water-based colloids and that contain many other matrix components; and (3) adhesive proteins, such as FN and LN, that are used to bind to the stroma (21).

### Collagens

Excessive production of collagens is a common feature of breast fibrosis and malignant BC. Collagens are the most abundant proteins in animals, accounting for more than 30% of the total protein content in the human body. Collagen is found in various organs and tissues and serves as the ECM framework. It can be synthesized and secreted into the ECM by fibroblasts, chondrocytes, osteoblasts, and some epithelial cells. At least 28 different types of collagen have been found; they are encoded by different structural genes and have various chemical structures and immunological properties (22, 23). The composition of collagen changes significantly in BC, with increased accumulation of type I, III, and V fibrillar collagens and decreased amounts of type IV collagen. The marked reduction of type IV collagen in BC is mainly due to basement membrane degradation (24). Studies have shown that certain collagen genes expressed in patients with primary BC are associated with an increased risk of metastasis. Increased expression of fibrillar collagen (e.g., type I and type III collagen) in BC may be associated with tumor invasion and aggressive tumor behavior. The relationship between changes in collagen production and BC progression may be functionally important (25–27).

The structural and physical properties of the ECM are continually changing in BC (28). Collagen type I is usually highly linearized, unlike the nondirectional fibrils. Linearized collagen type I is aligned along the epithelium or vertically into the tissue. Collagen can be used as a scaffold to facilitate the migration of cancer cells or stromal cells. Consistent with these observations, malignancy-associated fibrotic reactions, called fibrous hyperplasia, have been associated with poor prognoses in patients with BC. Collagen in the ECM of BC is heavily deposited in early cancer progression and plays a major role in maintaining tissue hardness (the stroma of BC is 10 times harder than normal), thereby triggering signal transduction in noncancer and tumor epithelial cells (29).

Certain collagens processed in the ECM are catalyzed by specific proteases, and the expression of enzymes associated with ECM remodeling is often upregulated in human cancers (30, 31). In many cancers, heparanases, cysteine cathepsin, 6-o-sulfonase, urokinase, and matrix metalloproteinases (MMPs) have been overexpressed. Lysyl oxidase (LOX) and lysine hydroxylase catalyze cross-linking between collagen and elastin molecules, further altering cellular behavior by regulating the ECM elasticity and strength (32). Any type of modification or addition of a cross-linked matrix makes BC tissue stiffer and enhances cell growth, survival, and migration-related signaling pathways (33).

### Elastin

Elastin gives tissues the flexibility they need to stretch and return to their original state. This property is helpful to lungs, skin, blood vessels, and neck ligaments. Smooth muscle and fibroblasts contain much elastin. Tropoelastins are the precursors of elastin; they are bound to a chaperone when they are secreted; they are additionally modified when they come into contact with mature elastin fibers, when then chemically transform into elastin chains. Lack of elastin in the ECM can lead to cutis laxa and Williams syndrome (34–36).

Elastosis is generally believed to be caused by the abnormal increase in the expression of elastic fiber components such as elastin and the degradation of normal elastic fiber. Elastosis is a common feature in BC (37). Ductal elastosis is closely related to cancer, especially invasive cancer. Elastosis reportedly increases with increasing severity of breast disease (37). BC elastosis is a complex phenomenon resulting in both deposition of elastotic masses and local production of elastin fragments. These two manifestations must be distinguished within the matrix. Signals from the fragments of the degraded matrix differ substantially from those provided by their parent proteins. For example, stromal cells adhere to elastin but cause cell migration and/or proliferation when they are incubated with elastin fragments (38).

Studies have shown that elastin-derived peptides have numerous biological activities in cancer cells and their surrounding stroma (36). They enhance tumor cell migration and stromal invasion (39, 40). Elastin-derived peptides also stimulate the migration and proliferation of skin fibroblasts and monocytes. They upregulate the expression of MMPs through a fibroblast-induced remodeling program, thus facilitating the invasion of melanoma cells (41, 42). In addition, they promote angiogenesis, chemotaxis for inflammatory cells, and the release of elastase. Finally, elastin-derived peptides provide a powerful survival signal, because they promote resistance to apoptosis (43, 44).

### Glycosaminoglycans and Proteoglycans

GAGs are unbranched long-chain polysaccharides composed of repeating disaccharides. The disaccharide unit usually consists of amino hexose (glucosamine or glucosamine galactose) and uronic acid; however, in keratinic sulfate, the uronic acid is replaced with galactose (45). Hyaluronic acid (HA) is the only aminoglycan that does not undergo sulfation, and its sugar chain is extremely long. Aminoglycans generally consist of fewer than 300 monosaccharides, whereas HA may contain 100,000 glycosyl groups. HA molecules in solution are in an irregular, curled state; if forced to stretch, their length can reach 20 m. The entire molecule consists of the repeated arrangement of glucuronic acid and acetylglucosamine disaccharides. HA can bind numerous water molecules because of the large number of negatively charged hydrophilic groups on its surface. Even at a very low concentration, it can form a viscous gel that occupies a large space and produces turgor. HA forms a significant part of the ECM and plays many biological roles in physiological and pathophysiological processes, depending on the size of its polymer and its interaction with other secreted proteins and cellular receptors (46, 47).

HA is involved in cancer progression and is remarkably increased in BC versus in normal breast tissue (48). Clinically, HA may be associated with highly invasive BC. Serum HA levels in patients with metastatic BC were significantly higher than those in patients without metastatic BC (49). HA regulates a variety of cellular behaviors, such as adhesion, growth, motility, and differentiation, and acts through surface receptors, such as the HA-mediated motor receptor (50) and CD44 (51). Hyaluronan synthase (HAS) is the key enzyme in HA biosynthesis (52). In animal models of BC, HAS2 expression promoted breast tumor progression and metastasis (53). Furthermore, in BC models, inhibition of HAS2 significantly reduced cancer progression, suggesting that it plays an important role in this process (54). Additionally, HA induces the invasive behavior of cancer cells themselves and causes increased hydration and interstitial pressure *via* CD44, which promotes fibroblast penetration as well as the migration and invasion activity of cancer cells (55).

### Laminin

LN is a large glycoprotein that, with collagen type IV, forms the ECM base. LN is involved in the embryonic development of ECM components at the earliest phase. LN molecules have at least eight binding sites. For example, the IKVAV pentapeptide sequence on the chain binds to neuronal cells and promotes nerve growth. The RGD sequence on the first chain of rat LN can bind to αvβ3 integrins (5, 56). LN has high sugar content (accounting for 15%–28%), and it has approximately 50 sugar chains connected with N-terminal. It is the most complex glycoprotein with a sugar chain structure identified so far. Moreover, the multiple receptors of LN recognize and bind its sugar chain structure. The basement membrane is the soft, specialized ECM under epithelial cells that also surrounds muscle, fat, and Schwann cells. It protects and filters cells, and it determines their polarity, which affects cell metabolism, survival, migration, proliferation, and differentiation (57).

Several LN subtypes play important roles in the development of BC. These subtypes include LM-111, LM-332, and LM-511 (58–60). LM-111, an important component of the basement membrane, is secreted by normal breast myofibroblasts and maintains epithelial cell polarity. LM-111 promotes prolactin-induced mammary epithelial cell maturation. In breast tumors, LM-111 expression is often lost, which results in changes in cell polarity. Studies have shown that LM-111 can induce cell–cell adhesion, suggesting that LM-111 may inhibit the spread of BC (61). There is growing evidence that other laminins containing α4 subunits, such as LM-332 and LM-511, may promote tumor progression. Expression of LM-332 is associated with aggressive BC, and cancer-derived LM-332 has promoted anchor-independent survival by interacting with α6β4 integrin receptors (62). In addition, LM-332 has induced migration and invasion of BC cells through α3 integrin. LM-511 has mediated adhesion, migration, and invasion *in vitro* and metastasis *in vivo* through integrin interactions in experimental models of BC. LM-511 interacted with the integrin α6β1 receptor in a subpopulation of BC cells capable of self-renewal and tumor initiation (63).

### Fibronectin

FN is a large glycoprotein found in all vertebrates; it has a molecular sugar content of 4.5%–9.5%. The sugar chain structure varies according to the origin and differentiation state of the tissue’s cells (64). FN connects cells to the ECM. Some of the short peptide sequences in FN are the smallest structural units for recognizing and binding to FN receptors on the cell surface. For example, the RGD (ARG-gly-ASP) sequence exists in the cell-binding module at the center of the peptide chain, where cell surface integrin receptors recognize and bind to them. FN molecules on the cell surface and in the ECM are cross-linked by disulfide bonds and assembled into fibers (65). Unlike collagen, FN does not self-assemble into fibers. Instead, it is directed by cell surface receptors that exist only on the surface of certain cells (e.g., fibroblasts). The decrease or loss of FN fibers on the surface of transformed cells and tumor cells is due to the abnormality of FN receptors on the cell surface (66, 67).

In cancer, FN is expressed by cancer-associated fibroblasts (CAFs) and by the cancer cells themselves (68). The upregulation of FN in cancer cells may occur through different mechanisms. Mechanical compression can lead an over expression of FN in cancer cells and increased invasion and migration behavior in tumors (69, 70). In general, FN expression in BC is associated with adverse clinical outcomes. Some studies have shown that cancer-derived FN is particularly associated with poor outcomes in BC, including increased metastases and reduced overall survival (70). In addition, FN expression has been detected in circulating tumor cells of patients with BC. Evidence from samples from patients with BC suggests that circulating tumor cells may have the property of epithelial-mesenchymal transformation; as a result, loss of cell polarity and cell–cell adhesion are observed in the epithelium, and a mesenchymal phenotype with high motility is acquired. FN is an established mesenchymal marker, and it has promoted transforming growth factor β–induced epithelial-mesenchymal transformation (25).

## ECM Remodeling Enzymes

During tissue regeneration and cancer, changes in the ECM also affect remodeling enzymes (18). Multiple ECM remodeling enzymes that promote stem/progenitor cell signaling pathways and metastasis are induced in BC (71). The process of ECM reconstruction in BC involves different signaling pathways of ECM regulation, including Wnt, PI3K/Akt, extracellular signal-regulated kinase, jun N-terminal kinase, Src-focal adhesion kinase (FAK), and others (20). The main induced proteins in the ECM are fibrinogen, proteoglycans, and matricellular cell proteins—all of which may be potential drug targets (29, 72). ECM remodeling enzymes such as MMPs, heparanase, urokinase plasminogen activator, cross-linking enzymes of the LOX family, and cathepsin, are often upregulated in breast tumors and have great significance to BC progression and metastasis (73–76). These enzymes modify the ECM in different ways to enable pathways of minimal resistance that promote cancer cell invasion and migration (18, 19). They can directly affect the biological properties and functions of ECM components by exposing cryptic sites or releasing ECM-bound growth factors or soluble domains of ECM proteins. Moreover, remodeling enzymes can change the physical properties of ECM structures by means of cross-linking and other modifications. These functions promoted by ECM remodeling enzymes are critical for BC progression and metastasis.

## Treatment Resistance Induced by ECM

The ECM in breast tumors is integral to the invasion and metastasis of BC. In recent years, increasing evidence has indicated that the ECM may also play an important role in mediating resistance to existing treatments (77–79). It provides an adhesion matrix and specific matrix components that promote survival mechanisms to support and induce stem cell pathways and enhance cell metastasis and invasion (80). Some researchers have found that BC matrix components are resistant to the chemotherapy drugs 5-fluorouracil (81), epirubicin (82), and cyclophosphamide (83). The ECM plays an important role in promoting resistance of cancer cells to treatment. Matrix proteins that induce chemotherapeutic drug resistance include the matricellular cell proteins, namely secreted phosphoprotein 1, tenascin C (84), B-lymphoma Mo-MLV insertion region 1, and phosphatase and tensin homolog (85). Hypoxia-induced ECM remodeling has been reported in BC (86). LOX is a key inducer of chemotherapeutic resistance. Inhibition of LOX can reduce collagen cross-linking and fibrinogen assembly, increase drug penetration, and downregulate ITGA5/FN1 expression, thereby inhibiting the Src/FAK signaling pathway and inducing apoptosis and chemotherapeutic resensitization. In addition, the ECM is associated with resistance to endocrine-targeted therapy and radiotherapy. Helleman et al. (2008) reported that ECM gene clusters were associated with resistance to first-line tamoxifen treatment in patients with metastatic BC. They indicated that expression levels of FN1, LOX, SPARC, and tissue inhibitor of metalloproteinase 3 were associated with prognosis in patients with BC, whereas tenascin C was associated with tamoxifen resistance (87).

Furthermore, the constitutive activation of PI3K as a result of both *PIK3CA* mutation and *PTEN* deletion are associated with resistance to human epidermal growth factor receptor 2–targeted therapy. This mechanism may explain the poor prognoses of some patients after trastuzumab treatment (88).

Finally, the ECM also promotes resistance to radiation therapy in BC. Resistance to single or combined drugs and radiation exposure, as mediated by cell adhesion, is driven by specific ECM proteins. Specifically, FN and LN enhance the resistance of human tumor cells and normal cells to ionizing radiation and cytotoxic drugs *in vitro* (89).

## Potential Therapeutic Targets in the ECM for BC

An increasing number of experimental studies have explored the biological roles of the ECM in BC and indicate that ECM components and ECM-mediated functions might be promising therapeutic targets (90–92). Many approaches are available to interfere with ECM function in BC. Chemical and biological agents that modulate the ECM in cancer are being investigated as potential treatments for BC (**Table 1**).

**Table 1:** Examples of potential therapeutic targets in the ECM of breast cancer.

Target	Therapeutic agent	Type	Effects on breast cancer	Ref.
HAS2, CD44	4-MU	Small molecule inhibitor	Inhibiting hyaluronic acid synthesis, regulating HAS2, CD44, matrix degrading enzymes, and inflammatory mediators	(93)
Robo1	R5	Neutralizing antibody	Significantly inhibited BC growth and metastasis MMTV-PyMT transgenic mouse model and xenografted breast cancer model	(91)
EphA2	CD44 exon V10	DNA aptamers	Significantly inhibited BC cell migration	(92)
MMP-2	MMPIs	Remodeling enzyme inhibitor	Specifically inhibited MMP-2 and prevent breast tumor growth and associated bone destruction	(94)
Heparanase	9E8, H1023	Monoclonal antibody	Significantly inhibited cell invasion and tumor metastasis, no significant cytotoxicity to BC cells	(95)
Zinc transporter LIV-1	SGM-LIV1A	Blocking antibody	SGN-LIV1A displays specific *in vitro* cytotoxic activity against LIV-1-expressing cancer cells; *in vitro* results are recapitulated *in vivo* with antitumor activity in animal models	(96)
(VCP)/p97	NPD8733	Small molecule inhibitor	NPD8733 silenced VCP expression in NIH3T3 fibroblasts and reduced the migration of the co-cultured NIH3T3 fibroblasts	(97)
Src/FAK	Lycorine	Small molecule inhibitor	Inhibited tumor growth in a breast cancer xenograft model and inhibited breast cancer metastasis in the MDA-MB-231 caudal vein model	(98)
TAMs	Endostatin	Recombinant peptide	Indicate the mouse breast cancer growth *in vivo* by regulating the polarization of tumor-associated macrophages	(99)
Endostatin	rh-Endostatin	Recombinant peptide	Chemotherapy combined with rh-endostatin is more effective than chemotherapy alone and is considered a promising breast cancer treatment strategy	(100)

HAS2, Hyaluronan synthase 2; 4-MU: 4-methylumbelliferone; EphA2, Erythrogenic human hepatocytes A; MMP-2, matrix metalloproteinase-2; MMPIs, MMP inhibitors; VCP, valosin-containing protein; Src/FAK, Src-focal adhesion kinase; TAM, tumor-associated macrophage; BC, breast cancer.

### Inhibition of ECM Components

Inhibiting ECM components that promote tumor progression and metastasis might be an attractive treatment strategy for BC (20). One possible approach is to suppress the synthesis of ECM components with pro-tumor functions. Preclinical studies in animal models have shown that this approach is feasible and can be translated into clinical practice (101–103). A recent study found that 4-methylumbelliferone can significantly reduce the migration, adhesion, and invasion of estrogen receptor cells in BC and reduce the expressions and activities of several pro-tumor matrix-degrading enzymes and pro-inflammatory molecules. The results suggest that 4-methylumbelliferone could be a new treatment for specific BC subtypes according to estrogen receptor status by inhibiting the synthesis of HA and regulating HAS2, CD44, matrix-degrading enzymes, and inflammatory mediators (93). Direct neutralization of tumor-causing ECM components is an attractive approach. Recent preclinical models have yielded encouraging results, showing that ECM components can be directly blocked by targeted peptides, neutralizing antibodies, or DNA aptamers (19). For example, Li et al. (91) found that R5 (a neutralizing antibody to Robo1) significantly inhibited BC growth and metastasis in an MMTV-PyMT transgenic mouse model and in a xenografted BC model. Lida et al. sed the SELEX (systematic evolution of ligand by exponential enrichment) method to develop DNA aptamers that specifically recognize the CD44 exon V10. These special aptamers significantly inhibited BC cell migration. The pull-down analysis indicated that the exon interacted with EphA2, which plays a key role in promoting tumor invasion and metastasis; this interaction was inhibited by the aptamers. The results of this study suggest that a novel molecular complex composed of CD44 and EphA2 can promote the progression of triple-negative BC (92).

### Targeting the ECM Remodeling Enzymes

Collagen cross-linking and ECM remodeling enzymes play vital roles in the development of rigid fibrotic tissue. They promote BC progression and metastatic spread. Antifibrotic therapies that target ECM remodeling enzymes may represent a good cancer treatment strategy (19).

MMPs are considered promising targets for cancer treatment. Numerous evidence indicates that MMPs play important roles in tumor invasion, metastasis, and angiogenesis; these findings have led to the development and clinical application of MMP inhibitors (MPIs). The new generation of MPIs is more selective for pre-metastatic MMPs and is currently being developed and tested in preclinical cancer models (104–107). In different models, tumor necrosis and apoptosis were induced by drug administration alone or in combination with chemotherapy. The clinical applications of many MPIs are not ideal because of their high musculoskeletal toxicity, poor bioavailability, low selectivity, or lack of efficiency (108). The conclusion from early trials was that inhibitor specificity was the key problem. Devel et al. optimized S1′ pocket interactions and recruited phosphinate or P2′ glutamate as alternatives to more traditional zinc-chelating groups, resulting in very selective MMP-12 inhibitors (109–111). A unique approach to limiting MPI toxicity was recently reported in a study aimed at achieving selectivity not only to the target enzyme but also through targeted drug delivery. Although MMP-2 is not generally considered a drug target in cancer because of its ubiquitous presence and involvement in many physiological processes, the importance of this enzyme in the progression of bone-metastatic BC suggests that it may be a useful target for this particular environment (94). Tauro et al. investigated phosphonic acid–based inhibitors selective for MMP-2 instead of the similar MMP-9. Because of the affinity of bisphosphonate for hydroxyapatite, these MPIs efficiently localized in the bone microenvironment. In a mouse model, the MPIs specifically inhibited MMP-2 and prevented breast tumor growth and its associated bone destruction (94).

Experimental evidence suggests that inhibiting heparanase may inhibit BC growth and metastasis. Therefore, drugs that have this effect may be feasible treatments for breast tumors. Several carbohydrate-based heparanase inhibitors have entered clinical trials. These compounds are highly similar to enzymes and work in combination with the heparin/Heparan sulfate (HS) -substrate binding domain of the enzyme, thus blocking their accessibility to the natural HS substrates (112). Weissmann et al. designed two monoclonal antibodies (9E8 and H1023) that neutralized the enzymatic activity of heparanase and significantly inhibited cell invasion and tumor metastasis without significant toxicity to BC cells themselves. This result suggests that monoclonal antibodies affect the tumor microenvironment rather than the BC cells, thus offering a newer and safer mode to obstruct both tumor growth and metastasis (95).

### Antibodies Against Remodeled ECM

Certain ECM components are highly expressed in areas of active tumor invasion and thus can be used as biomarkers. Anti-remodeling ECM antibodies may introduce bioactive-inhibiting cues or radioactive substances into tumor sites. This approach can enhance the effectiveness of radiation, chemotherapy, or targeted therapy by concentrating radioisotopes, drugs, or antitumor biologics on the active tumor sites while minimizing their distribution in healthy tissue (113–116). Huang et al. enhanced the potency of a whole-cell BC vaccine in mice with an antibody–interleukin (IL)-2 immunocytokine that targets exposed phosphatidylserine. Immune cytokines (2AG4-IL2) were prepared by linking IL-2 to the 2aG4 gene, a targeted antibody that blocks the immunosuppressive effects of phosphatidylserine. The 2AG4-IL2/4T1 vaccine was prepared after phosphatidylserine-exposed, irradiated 4T1 cells were coated with 2AG4-IL2. The incidence and number of spontaneous lung metastases in the 2AG4-IL2/4T1 group were significantly lower than those in other groups. Spleen cells of mice immunized with 2AG4-IL2/4T1 showed significantly higher 4T1-specific cytotoxicity and demonstrated a greater ability to secret interferon-γ than spleen cells of other groups. These results suggest that 2AG4-IL2 encapsulation of radioactive tumor cells can produce an effective whole-cell vaccine (117). Sussman et al. investigated a novel antibody-drug conjugate (ADC; SGN–LIV-1A) that targeted the zinc transporter LIV-1 (SLC39A6) for the treatment of metastatic BC. LIV-1 is expressed by estrogen receptor–positive BC. The data on the latest ADCs and their recent successes support the use of SGN–LIV-1A as a new treatment for refractory metastatic BC and other LIV-1–positive indications (96).

### Cell Surface Receptor Blockers

Cell surface receptor blockers that interact with ECM components may be a way to treat metastatic BC. Interactions between cells and ECM proteins are often mediated by integrins. Integrins belong to the transmembrane protein family and act as cell surface receptors that mediate cell-to-cell and cell-to-matrix adhesion by identifying components of ECM, such as FN, laminin, collagen, and transmit information from ECM to cells (118), leading to the recruitment and activation of intracellular signaling proteins, which in turn initiates a signaling cascade that promotes breast cancer cell migration, proliferation, and survival (119, 120). The increase of integrins will facilitate the tumor cells adhesion to the basement membrane and promote the invasion and metastasis of tumor cells. Therefore, integrins represent an interesting target for metastatic BC therapies. In addition, recent researches in the xenograft mouse model showed that surface integrin α2β1 contacts and activates Wnt-β-catenin. The integrin α2β1 plays a pro-metastatic role in BC progression, and integrin α2β1–silencing has a potential effect in inhibiting breast cancer metastasis (121, 122). A study conducted by Archis Bagati et al. indicated that SOX4 expression is regulated by the integrin αvβ6 receptor on the tumor cell surface, which activates TGFβ from a potential precursor. They identified that SOX4 transcription factor acts as an important T cell-mediated cytotoxic resistance mechanism of TNBC. An integrin αvβ6/8-blocking monoclonal antibody (mAb) inhibits SOX4 expression and sensitizes TNBC cells to cytotoxic T cells. In a highly metastatic mouse model of TNBC, this integrin mAb induced a substantial survival benefit (123).

### Targeting Cancer Associated Fibroblasts

CAFs are the major players in dysregulation of collagen transformation during tumor progression, leading to tumor fibrosis characterized by excessive depositions of collagen around the tumor (124). Unlike normal fibroblasts, CAFs overexpress a number of biomarker proteins. Generally speaking, the interaction between CAFs and tumor cells promotes tumor progression mainly through the release of various secreted proteins (e.g., transforming growth factor-β, insulin-like growth factor, and IL-6), direct interaction with tumor cells, immune response regulation, and ECM remodeling. These multiple actions suggest that several stromal therapeutic targets exist (25, 125, 126). Suvarna et al. demonstrated that NIH3T3 fibroblast migration was enhanced after co-cultured with MCF7 BC cells. They found that a small-molecule ligand (NPD8733) of valosin-containing protein/p97 inhibited cancer cell–accelerated fibroblast migration. This mechanism offers potential for BC treatment (97). Many activated CAFs are present in the BC stroma, and they have the ability to promote tumor formation and development. Studies have shown that CAFs are the main factor involved in ECM remodeling (127, 128). Activated CAFs can specifically change some ECM components, transforming the ECM from loose and irregular to linear, and driving changes in biomechanical conduction (69). Cell polarity changes when cancer cells perceive changes in extracellular stress; invasion and metastasis. The mechanism by which CAFs remodel the ECM and thus affect the function of cancer cells has been discovered gradually. CAFs come from numerous sources, especially from normal fibroblasts (70). The transformation of normal fibroblasts to CAFs is a process of cell differentiation, and miRNA plays an important role in embryonic development (129–132). A Study has shown that miR-200 mediates the differentiation of normal fibroblasts into CAFs by regulating the expression of cell differentiation–related transcription factors, TCL12 and FLI-1, and miR-200 maintains the activation state of CAFs. The continuously activated CAFs directly or interactively regulate the expression of ECM-related genes, participate in the remodeling of ECM, and further promote the occurrence and development of breast tumors (71).

### Targeting Endostatin

Some ECM cues suppress tumor and metastasis development, so enhancing these biological effects may offer a feasible approach to BC treatment (19). Angiogenesis plays a critical role in the pathogenesis, growth, invasion, and metastasis of solid tumors. Endostatin, which was first isolated in 1997, is one of the most effective anti-angiogenic factors and has significantly reduced blood vessel formation in tumors (133). Guo et al. demonstrated that endostatin induced RAW264.7 phenotype polarization to M1 *in vitro*. They proposed that endostatin may inhibit mouse BC growth *in vivo* by regulating the polarization of tumor-associated macrophages *via* two possible mechanisms: by shifting the polarity of the tumor-associated macrophage from an M2-like to an M1-like functional phenotype or by increasing the proportion of M1-like tumor-associated macrophage *via* specific inhibition of M2 polarity (99). Chen et al. conducted a phase III, multicenter, prospective, randomized, controlled clinical trial to explore the efficacy and safety of rh-endostatin. Patients received neoadjuvant therapy with docetaxel and epirubicin or that combination plus rh-endostatin. After three cycles of neoadjuvant therapy, the objective effective rates of the docetaxel and epirubicin group and of rh-endostatin group were 77.9% and 91.0%, respectively (p < 0.001). Chemotherapy combined with rh-endostatin is more effective than chemotherapy alone and is considered a promising treatment strategy for BC (100).

## Novel Nanoparticle-Based Approaches to Modulate ECM Components in BC

The strategies for ECM-based therapy have been attractive in recent years, so many drugs are emerging to target the ECM. Currently, targeted drug delivery systems can effectively solve the problems conventional chemotherapy caused. They can preferentially deliver drugs to tumor tissue, thus preventing the dose-limiting adverse effects that occur in normal tissues (11–13, 134). Several nano-based therapies targeting the ECM have been used for tumor theranostics. Some of these NPs are single drug carriers, but many more are drugs, genes, antibodies, or aptamers combined; some have multiple functions, such as diagnostics, therapeutics, and monitoring. These emerging nanotherapeutics that can regulate the ECM have roughly three approaches: (1) degrading the tumor ECM; (2) mimicing tumor ECM to inhibit tumor progression; and (3) intervening the native ECM fabrication. The first is aimed at breaking barriers to improve tumor penetration of the nanomedicine delivery system; the second is aimed at enhancing measures to block tumor metastasis in the early stages of tumor progression; and the last is aimed at specific stages in the body when the tumor’s ECM is made. This review presents the recent developments of NPs in ECM-targeted therapy (**Table 2**).

**Table 2:** Overview of selected nanoparticles that target the breast cancer ECM.

Application	NPs	Characteristics and functions	Ref.
Degrading the tumor ECM	Gold NPs	Through the interaction between the coated gelatin layer and MMP-2 in the ECM, large-sized gold NPs become smaller, enabling deeper tumor infiltration	(135)
		Gold NPs combined with MMP-sensitive peptides can be employed in drug delivery and tumor imaging	(136)
	Liposomes with GPLPLR peptide	A GPLPLR peptide sequence modified to target MT1-MMP was more effective in binding and treating tumors than uncoated liposomes	(137)
	Collagenase-encapsulated polymers	Released CLG enzyme can specifically degrade collagens, leading to a loosened ECM structure, enhanced tumor perfusion, and less hypoxia	(138)
Simulating tumor ECM	Transformable laminin (LN)-mimic peptide	Efficiently inhibited lung metastasis in breast and melanoma tumor models	(139)
	Dual-degradable and injectable hyaluronic acid hydrogel	Expression levels of VEGF, IL-8, and bFGF in hydrogel-cultured cells were significantly greater than those in 2D culture	(140)
Intervening the native ECM fabrication	LOXab-NPs	LOXab-NPs are highly specific for tumor targeting in xenograft models	(141)
	pH-sensitive cleavable liposomes	Depletion of collagen I by PTX-Cl-Lip and the combination of free losartan and PTX-CL-Lip could enhance the antitumor efficacy of chemical drugs	(142)
	DOX-AuNPs-GNPs	Pretreatment with losartan significantly decreased collagen levels and improved tumor penetration	(143)

ECM, extracellular matrix; NPs, nanoparticles; LOXs: lysyl oxidase family; MMP-2, matrix metalloproteinase-2; PTX, paclitaxel; VEGF, vascular endothelial growth factor; IL-8, interleukin 8; FGF, fibroblast growth factor; DOX, doxorubicin.

### Nanoparticle Based ECM Degradation

**Figure 1:**
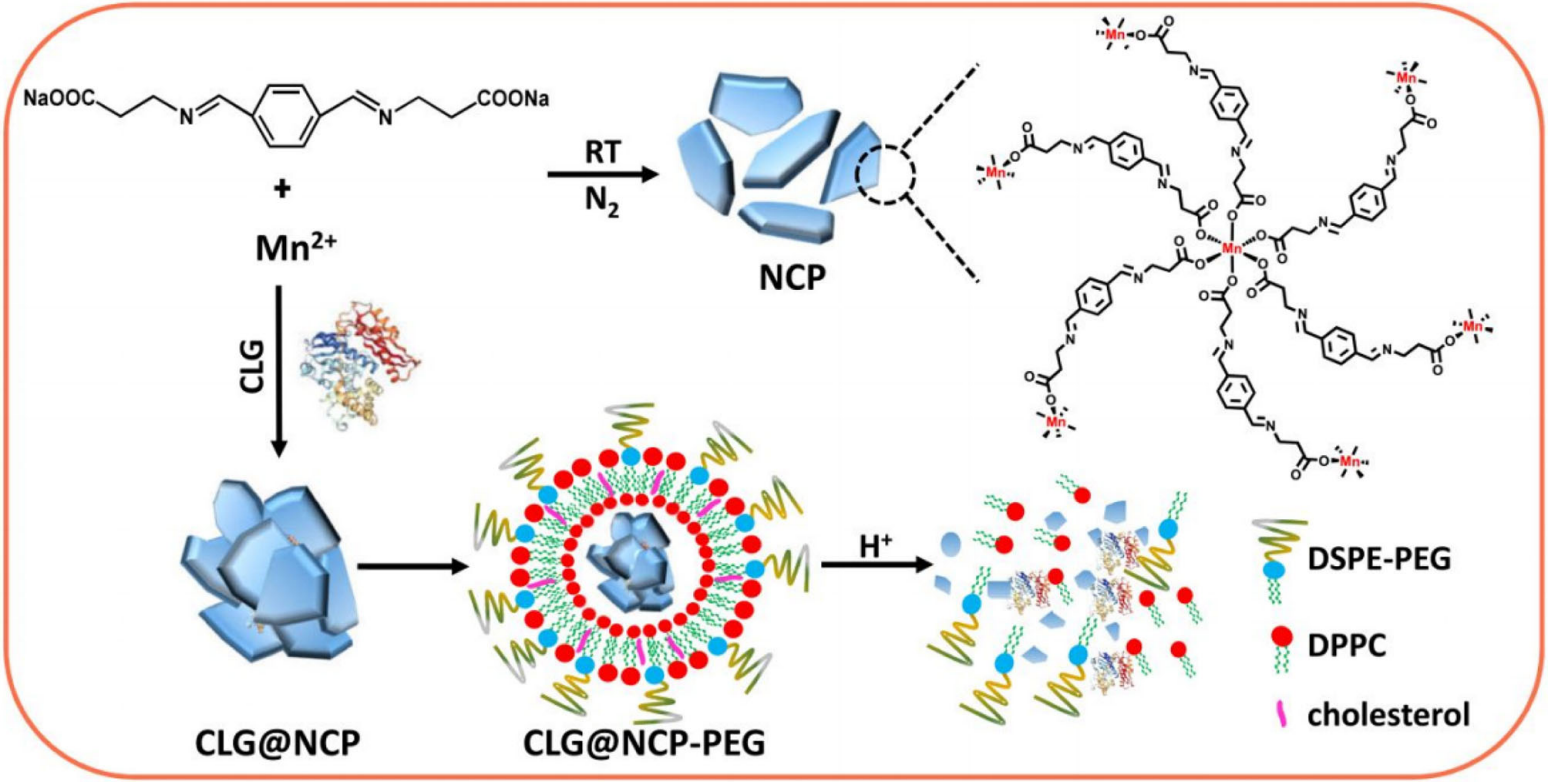
Synthesis and characterization of CLG@NCP-PEG NPs. Scheme for the preparation of NCP NPs and CLG-encapsulated CLG@NCP nanoparticles, surface modification, and pH-sensitive degradation. Reproduced with permission from (138).

The cleavage of ECM components depends on the balance of proteases and protease inhibitors, which promotes the release of bioactive molecules. In particular, MMPs and tissue inhibitors of metalloproteinases play important roles in controlling the ECM (13). The strategy to degrade ECM components and increase NP affinity is widely used. Because NPs cannot reach deep-seated tumors *via* conventional methods, many contractible NPs have been designed to penetrate tumor vasculature and tissue. Through the interaction between the coated gelatin layer and the MMP-2 in the ECM, large-sized gold NPs become smaller, which enables deeper tumor infiltration (135). Kessenbrock et al. designed a liposome modified with a GPLPLR peptide sequence to target MT1-MMP, which was more effective than uncoated liposomes in binding and treating tumors (137). They also designed gold NPs combined with MMP-sensitive peptides for drug delivery and tumor imaging. These gold NPs have efficient drug delivery and imaging capabilities at tumor sites. Suresh et al. (136) found that MMP-2–sensitive gold NPs improved targeted delivery to human BC cells and increased cellular uptake. The proteases collagenase and hyaluronidase are also commonly used to control ECM degradation. For example, Liu et al. synthesized collagenase-encapsulated nanoscale coordination polymers based on Mn^2+^ and an acid-sensitive benzoic-imine organic linker that was then modified by polyethylene glycol (PEG). The results indicated that released collagenase could specifically degrade collagens, leading to a loosened ECM structure, enhanced tumor perfusion, and decreased hypoxia (**Figure 1**) (138).

### Mimicing the Tumor ECM to Inhibit Tumor Progression

Tumor metastasis refers to the formation of a new nidus at a distant site from the original tumor; it is the main reason for the high cancer-associated mortality rate. As mentioned above, the tumor ECM is critical to progression, and it provides an important protective layer to prevent tumor metastasis. Tumor cells overexpress some enzymes, such as MMP, to overcome ECM barriers and prepare for tumor metastasis. Many scientists have proposed mimicing the tumor ECM to inhibit tumor metastasis, and some promising results have been achieved (144–146). For example, Suo et al. (140) designed a dual-degradable and injectable HA hydrogel to mimic the ECM for a 3D culture of MCF-7 BC cells.

**Figure 2:**
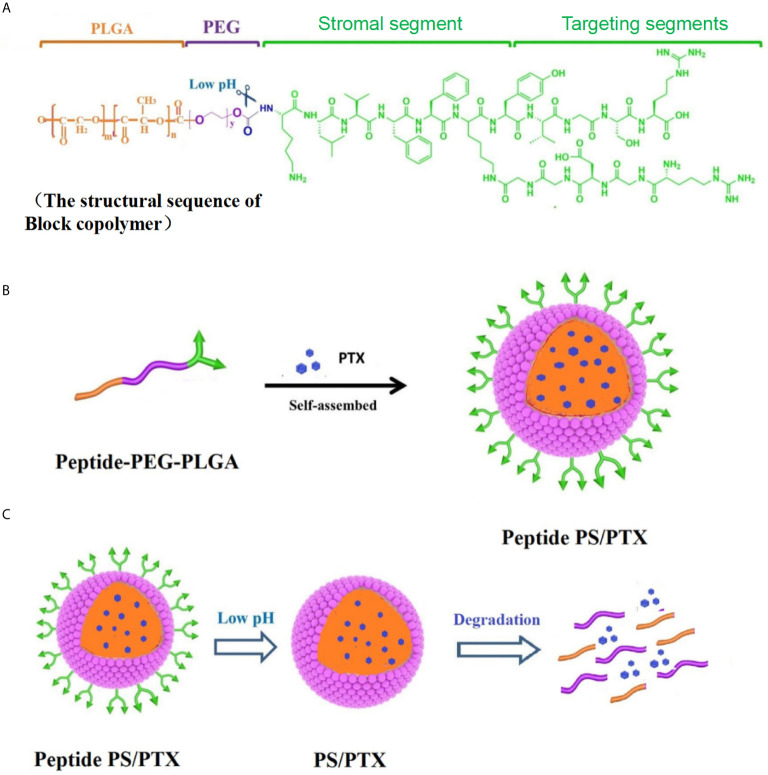
Synthesis and characterization of Peptide PS/PTX micelles. **(A)** Scheme of the lock copolymer structural sequence. **(B)** Peptide-PEG-PLGA conjugate is mixed with PTX and self-assembled into Peptide PS/PTX micelles. **(C)** Surface-modified peptide PS/PTX micelles and is pH-sensitive and glutathione-sensitive degradation.

Inspired by the natural ECM formation, our laboratory constructed a “fibrin integrin receptor” structural peptide (AANL-KLVFFK-GGDGR-YIGSR) to initiate and participate in the physiological reconstruction of the ECM in BC. LN is one of the most important ECM components (147); the YIGSR sequence in the LNβ1 chain and the RGD sequence in the LNα chain are considered essential to integrin identification. They also combine specific loci, including metal ions (Ca^2+^, Mg^2+^) adhesive dots, and produce the mechanical force behind cell actin filaments during ECM self-assembly. YIGSR targets integrin’s RGD receptor. In normal cells and on a mature endothelial cell surface, integrin α_v_β_3_ expression is low, but it is highly expressed during tumor angiogenesis (148). The drug delivery system can specifically bind tumor cells that express integrins, effectively mediate the drug delivery system to enable tumor cell entry, reduce distribution in normal tissue, and prevent fibrosis of the normal tissue cell matrix. In addition, we are currently synthesizing a peptide-PEG-PLGA three-block conjugate to obtain biodegradable polymer micelles through self-assembly. These novel NPs comprise three units: (1) YIGSR and RGD targets to induce nanomicelle transfer to the tumor site; (2) enzyme-sensitive groups of peptide-PEG-PLGA, under the action of a legumain, to easily dissociate in a tumor pH (pH 4–6.5) (148); and (3) KLVFF peptides that participate in ECM remodeling, curing the tumor tissue protein fiber layer. Then, after PLGA-PEG/PTX enters the cell, glutathione dissolves the disulfide bond between PLGA and PEG to release PTX. This release inhibits tumor cell tubulin depolymerization and promotes cell apoptosis in BC (**Figure 2**; data to be published).

### Intervening in Native ECM Fabrication

With a better understanding of the natural assembly process of the native tumor ECM, it is now possible to interfere with the specific stages of ECM fabrication in a tumor. An anti–LOX-like 2 antibody can alter the natural assembly of endogenous fibrous collagen. LOX-like 2 interference with collagen can significantly affect the adhesion and invasion of human BC cells, and tumor growth can be effectively inhibited by LOX-like 2 antibody treatment in mice bearing mammary tumors (149–151). PLGA-based LOX-traceable NPs consisting of LOX antibodies and paclitaxel were successfully synthesized and can be used as a tumor-targeting drug carrier for chemotherapy and targeted radiotherapy. LOX antibodies have high specificity to target tumors in xenograft models (141). These NPs can affect ECM fabrication in tumors by altering collagen assembly; in addition, they can increase drug concentrations in tumor radiotherapy sites and enhance the chemotherapeutic effect of paclitaxel, which achieves the same result as the method described in 6.2.

## Discussion

BC metastasis is a complex process with different regulatory mechanisms that involve many processes, including changes in the ECM (74). The ECM is a proven barrier to cells. During the process of tumor invasion and metastasis, a series of dynamic changes occurs in tumor cells and the surrounding ECM (19). These events involve MMPs (e.g., MMP-2 and MMP-9), cathepsin, fiber protease release, type IV collagen, LN, gelatin, fiber connection proteins, other components outside the matrix involved in synthesis and degradation, collagen enzyme inhibitors (e.g., tissue inhibitors of metalloproteinase 1 and 2), adhesion molecules, and tumor cells that release fibroblast growth factor, vascular endothelial growth factor, and angiogenesis inhibitors (20). Greater insight into the occurrence of metastasis and its mechanisms in BC, as well as a better understanding of ECM structure and regulation, will help spur the development of new therapeutic targets for antineoplastic drugs (12, 13, 146).

NPs offer an innovative technology with great potential for biomedical applications. A growing number of studies has demonstrated that ECM-targeted NPs hold great promise for early BC diagnosis and treatment. Nanomaterials are useful vehicles for improving anticancer drug efficacy. New advances have been made in the study of multifunctional NPs that provide simultaneous targeting of both tumor cells and the ECM (152, 153). However, many challenges remain in designing effective nanodrugs for clinical cancer treatment. For example, the interaction between the ECM and tumor cells is still largely unknown. Appropriate experimental and preclinical models must be designed to better describe the ECM and accelerate the development of existing NP systems. In addition, many problems in tumor metastasis and drug resistance urgently need solved. An immunosuppressive ECM impedes the implementation of effective immunotherapies (154). These cancer therapy obstacles require an advanced NP system that can precisely target the molecular determinants of the ECM. Future research should focus on developing NP systems that regulate ECM at the metabolic and immune levels; such a system would likely yield great clinical achievements.

## Conclusions

This review focused on the role of the ECM in the development and metastasis of BC and on the application of NPs that target this structure. The ECM composition dynamically changes in BC and is very different from that of a normal mammary gland. Various ECM-modifying enzymes play an important role in BC progression and metastasis. Moreover, the ECM may be involved in regulating resistance to treatments, including chemotherapy, endocrine targeted therapy, and radiotherapy. The ECM provides many potential therapeutic targets, and NPs are promising carriers for improving drug activity and efficacy. Additional research is needed to promote the development of ECM-targeted nanotherapeutics that effectively control BC tumors.

## Author Contributions

YCZ and XZ drafted the manuscript. CZ conceptualized the study. YQZ assisted in reviewing literature. The manuscript was modified by YC and WF. FW helped review the first draft of the manuscript. The final version was reviewed and edited by YCZ, XZ, and CZ. All authors contributed to the article and approved the submitted version.

## Funding

This work was financially supported by the National Natural Science Foundation of China (nos. 81802630, 81802587, 81873838) and Public Welfare Technology Research Project of Zhejiang Province (LGF21H160023).

## Conflict of Interest

The authors declare that the research was conducted in the absence of any commercial or financial relationships that could be construed as a potential conflict of interest.

## Abbreviations

ADC, antibody-drug conjugate; BC, breast cancer; CAF, cancer-associated fibroblast; ECM, extracellular matrix; FAK, Src-focal adhesion kinase; FN, fibronectin; GAG, glycosaminoglycan; HA, hyaluronic acid; HAS2, hyaluronan synthase; IL, interleukin; LN, laminin; LOX, lysyl oxidase; MMP, matrix metalloproteinase; MPI, MMP inhibitor; NP, nanoparticle; PEG, polyethylene glycol.
